# Editorial: Recent advances of metal-film electrodes for trace electrochemical analysis

**DOI:** 10.3389/fchem.2022.973672

**Published:** 2022-07-22

**Authors:** Vasko Jovanovski, Klodian Xhanari, Matjaž Finšgar

**Affiliations:** ^1^ Laboratory for Electrocatalysis, Department of Materials Chemistry, National Institute of Chemistry, Ljubljana, Slovenia; ^2^ Department of Chemistry, Faculty of Natural Sciences, University of Tirana, Tirana, Albania; ^3^ Faculty of Chemistry and Chemical Engineering, University of Maribor, Maribor, Slovenia

**Keywords:** metal film electrodes, stripping analysis, voltammetry, alloys, electrode modification, electroanalysis, analytical electrochemistry, trace electrochemical analysis

Metal-film electrode development started following and in parallel with mercury-based electrodes, like dropping mercury electrode (DME) and hanging mercury drop electrode (HMDE), the seminal work of 1959 Nobel Prize for chemistry winner J. Heyrovsky. Those electrodes have excellent electroanalytical performance. Scientists developed very sensitive methods for detecting trace level concentrations of basically all metals that form amalgams and furthermore, with the utilization of adsorptive stripping techniques, even those that do not form amalgams. Instead, the accumulation of metallic complexes and subsequent stripping leads to sensitive determinations of metals like Ni, Co and Cr. However, safe handling, measurements, and disposal of liquid mercury require appropriate infrastructure, knowledge and experience. In light of this, metal-film electrodes proved to be a viable and necessary alternative. Although with much less predictable geometry-mercury being in the shape of a smooth spherical droplet-metal film electrodes are still in use and development today. Many metals and alloys have been tested (including mercury film electrode) and have been showing good results for measuring trace levels of toxic metals, various electroactive organic compounds, gases (VOCs), biological assays, etc. Mercury alternatives, for example, gold, silver, iridium, bismuth, antimony, copper, etc., exhibited favourable electroanalytical characteristics, mainly for the determination of some of the most toxic metallic ions and in other aforementioned analytical applications. It has to be also mentioned that many of the metal-film electrodes do not require the removal of oxygen during measurements as mercury electrodes do. This further expedites the measuring procedure and makes it more viable for outdoor and on-site measurements.

Coinciding with the research primarily focused on the thin metal film electrodes was the development of new electroanalytical methods. Basic principles for measurements acquired from initial work with mercury electrodes were translated into the application of metallic films. With the introduction of differential pulse and square-wave voltammetry, the signal-to-noise ratio increased significantly. As a result, electrochemical techniques could become comparable to more sophisticated techniques such as AAS, ICP-MS, and ICP-OES, but with substantially lower costs for instrumentation and the measuring procedure itself. Current potentiostats offer low weight and thus portability, simple connectivity to laptops, tablets and smartphones, and in this way allow true portability for outdoor and remote sensing. Nowadays, novel electroanalytical methods are being developed based on a combination of “old” ones, taking advantage of such synergy. Novel methods like differential square-wave voltammetry and double sampled square-wave voltammetry, pioneered by Prof. Mirčeski, either expedite the measurement procedure, give more information about the electrochemical processes, or further increase the signal-to-noise ratio.

In this Research Topic in Frontiers in Chemistry entitled: *Recent Advances of Metal-Film Electrodes for Trace Electrochemical Analysis*, we can find a minireview by Wygant and Lambert. In this minireview the authors delve into the development and use of *in-situ* prepared thin-film modified electrodes in a variety of different applications. The authors have first briefly explained the preparation of such electrodes *via* simultaneous deposition onto the surface of a substrate electrode of several metals (e.g., Hg, Bi, Sn, etc.) alongside the analyte species. The analyte(s) can then be stripped, identified, and quantified from the *in-situ* prepared modified electrodes. Next, the authors discuss the application of thin-film ASV electrodes, which are found to be suitable for detecting the analyte(s) in both acidic and alkaline media, over a wide range of pH. The detection of trace levels of toxic metals in the environment, the characterization of battery systems and energy applications, as well as the analysis of biological assays and other medical applications, are among the most commonly reported applications of such electrodes. Finally, the authors discuss on future development and applications of these electrodes in ASV measurements.

In their research work, Zhou et al., feature the development of a silica nanochannel array film (SNF) coated β-cyclodextrin-graphene (CDG) nanocomposite modified Au film electrode (AuF) electrochemical sensor with anti-fouling ability, for the determination of acetaminophen in complex samples. Because of their rich surface hydroxyls and 2D lamellar structure, CDG modified on AuF can serve as the nanoadhesive for compact binding SNF, which can be grown by a fast electrochemical assisted self-assembly method. Attributable to the electrocatalytic property of graphene and the synergistic enrichment from both CDG and SNF nanochannels towards the analyte, the SNF/CDG/AuF electrochemical sensor demonstrates sensitive detection of acetaminophen ranging from 0.2 to 50 μM with a limit-of-detection of 14 nM. The anti-fouling ability of the developed electrochemical sensor enables accurate and convenient analysis of acetaminophen in commercially available paracetamol tablets.

In another research article, Zou et al., describe the development of a novel electrochemical aptasensor for diclofenac (DCF) determination. The fabrication of the aptasensor followed a two steps process. First, carboxylic acid–functionalized multi-walled carbon nanotubes (f-MWCNTs) were used to modify the glassy carbon electrode (GCE). Then, covalent immobilization of the amine-terminated DCF aptamer was performed on the surface of the modified GCE. The f-MWCNTs provide a reliable matrix for aptamer immobilization with high graft density, while the aptamer serves as a biorecognition probe for DCF. Electrochemical impedance spectroscopy (EIS) was employed to study the performance of the developed aptasensor based on the change in the charge transfer resistance (*R*_ct_) caused by the formation of a DCF-aptamer complex on its surface. The plot of *R*_ct_ vs. the logarithm of concentration displays two linear ranges for DCF detection from 250 fM to 1 pM and from 1 pM to 500 nM, with a detection limit of 162 fM. The reproducibility, acceptable stability, and reliable selectivity of the developed aptasensor were proven. The combination of its analytical performance with the simple design, and relatively easy modification procedure, makes the developed aptasensor of great interest in the trace determination of DCF in environmental applications.

In the last contribution, Fang and Duan, report on the development of an electrochemical sensor for capsaicin detection. Capsaicin was selectivity adsorbed in the cavities of a bimetallic MOF nanocage (Fe^III^-HMOF-5) used to modify a screen printed carbon electrode (SPCE). Sensing optimization was performed in terms of accumulation time and pH of the buffer. Under optimum conditions, the linear concentration range of the developed electrochemical sensor for capsaicin detection was found to be 1–60 μM. The detection limit of 0.4 μM was reported. The electrochemical sensor was used for capsaicin determination in the real sample, showing excellent stability and selectivity.

